# Antibacterial and Antivirulence Activity of Manuka Honey against Genetically Diverse Staphylococcus pseudintermedius Strains

**DOI:** 10.1128/AEM.01768-20

**Published:** 2020-10-01

**Authors:** Helen L. Brown, Georgie Metters, Matthew D. Hitchings, Thomas S. Wilkinson, Luis Sousa, Jenna Cooper, Harry Dance, Robert J. Atterbury, Rowena Jenkins

**Affiliations:** a Department of Biomedical Sciences, Cardiff Metropolitan University, Cardiff, United Kingdom; b School of Dentistry, Cardiff University, Cardiff, United Kingdom; c Swansea University Medical School, Swansea, United Kingdom; d Instituto de Ciências Biomédicas Abel Salazar, University of Porto, Porto, Portugal; e School of Veterinary Medicine and Science, University of Nottingham, Leicestershire, United Kingdom; INRS—Institut Armand-Frappier

**Keywords:** manuka honey, antibiotic resistance, synergy, hemolysis, proteolysis, aggregation

## Abstract

Staphylococcus pseudintermedius causes opportunistic infections in dogs. It also has significant zoonotic potential, with the emergence of multidrug resistance leading to difficulty treating both animal and human infections. Manuka honey has previously been reported to inhibit many bacterial pathogens, including methicillin-resistant Staphylococcus aureus, and is successfully utilized in both clinical and veterinary practice. Here, we evaluated the ability of manuka honey to inhibit strains of S. pseudintermedius grown alone and in combination with antibiotics, as well as its capacity to modulate virulence within multiple S. pseudintermedius isolates. All 18 of the genetically diverse S. pseudintermedius strains sequenced and tested were inhibited by ≤12% (wt/vol) medical-grade manuka honey, although tolerance to five clinically relevant antibiotics was observed. The susceptibility of the isolates to four of these antibiotics was significantly increased (*P* ≤ 0.05) when combined with sublethal concentrations of honey, although sensitivity to oxacillin was decreased. Virulence factor (DNase, protease, and hemolysin) activity was also significantly reduced (*P* ≤ 0.05) in over half of isolates when cultured with sublethal concentrations of honey (13, 9, and 10 isolates, respectively). These findings highlight the potential for manuka honey to be utilized against S. pseudintermedius infections.

**IMPORTANCE** Staphylococcus pseudintermedius is an important member of the skin microbial community in animals and can cause opportunistic infections in both pets and their owners. The high incidence of antimicrobial resistance in S. pseudintermedius highlights that this opportunistic zoonotic pathogen can cause infections which require prolonged and intensive treatment to resolve. Manuka honey has proven efficacy against many bacterial pathogens and is an accepted topical treatment for infections in both veterinary and clinical practice, and so it is a particularly appropriate antimicrobial for use with zoonotic pathogens such as S. pseudintermedius. Here, we demonstrate that not only is manuka honey highly potent against novel multidrug-resistant S. pseudintermedius isolates, it also acts synergistically with clinically relevant antibiotics. In addition, manuka honey modulates S. pseudintermedius virulence activity, even at subinhibitory concentrations. In a clinical setting, these attributes may assist in controlling infection, allowing a more rapid resolution and reducing antibiotic use.

## Abstract (precis)

Staphylococcus pseudintermedius is an important member of the skin microbial community in animals and can cause opportunistic infections in both pets and their owners. The high incidence of antimicrobial resistance in S. pseudintermedius highlights that this opportunistic zoonotic pathogen can cause infections which require prolonged and intensive treatment to resolve. Manuka honey has proven efficacy against many bacterial pathogens and is an accepted topical treatment for infections in both veterinary and clinical practice, and so it is a particularly appropriate antimicrobial for use with zoonotic pathogens such as S. pseudintermedius. Here, we demonstrate that not only is manuka honey highly potent against novel multidrug-resistant S. pseudintermedius isolates, it also acts synergistically with clinically relevant antibiotics. In addition, manuka honey modulates S. pseudintermedius virulence activity, even at subinhibitory concentrations. In a clinical setting, these attributes may assist in controlling infection, allowing a more rapid resolution and reducing antibiotic use.

## INTRODUCTION

Staphylococcus pseudintermedius is a commensal bacterium of the skin and mucous membranes of up to 80% of dog populations (1) and is frequently associated with opportunistic veterinary infections such as postsurgical infections and pyoderma (2, 3). Increasingly, methicillin-resistant S. pseudintermedius (MRSP) is being isolated from these infections, and reports now indicate that S. pseudintermedius isolates display resistance to a range of antibiotics, including erythromycin, clindamycin, ciprofloxacin, and gentamicin (4). This resistance to multiple classes of antibiotics has made S. pseudintermedius a global clinical challenge in veterinary medicine, as treatment of infections is increasingly problematic. The prevalence of S. pseudintermedius has implications that reach beyond the veterinary field, as studies have shown that S. pseudintermedius can cause zoonotic infections in humans, making it a significant health issue (5, 6).

The zoonotic potential of this organism has only recently been recognized because S. pseudintermedius infections in humans have previously been misidentified as Staphylococcus aureus due to their phenotypic similarities and coagulase positivity (7). These species share traits such as acquired methicillin resistance, ability to form biofilms, modulation of the host immune system, and production of proteolytic enzymes and toxins (8). Recent advances in diagnostics have allowed clearer separation of the two species and have shown that, as well as being a zoonotic agent, S. pseudintermedius is able to colonize the nasal passageways of humans. This colonization provides a reservoir for reinfection of both animals and humans, and a potential reservoir for antimicrobial resistance gene transfer (9). As S. pseudintermedius has exhibited a decreasing level of antibiotic susceptibility and can cause severe infection in both animals and humans, there are grounds for evaluating whether new strategies or novel antimicrobial agents could be used to enhance treatment options (10).

Manuka honey is used as a topical antimicrobial agent for infections in both humans and animals, exhibiting activity against a wide range of pathogens (11). Antimicrobial activity is due to the presence of multiple antimicrobial compounds within the honey, the best studied of which is methylglyoxal, together with a high osmotic potential (for a review of the activity of manuka honey, see reference 12). Manuka honey has been used to successfully eradicate S. aureus infections in the clinic and can inhibit the growth of methicillin-resistant S. aureus (MRSA) and Pseudomonas aeruginosa at low concentrations *in vitro* (13, 14). The mode of action of manuka honey against MRSA has been partially elucidated, and, due to the phenotypic similarity between S. aureus and S. pseudintermedius, is highly likely to be similarly effective in inhibiting S. pseudintermedius and MRSP (15). More recent studies have also highlighted the ability of manuka honey to increase efficacy of some clinically relevant antibiotics against MRSA, which could also be useful in the case of difficult-to-treat antibiotic-resistant S. pseudintermedius infections (15). In parallel, manuka honey has been utilized by the veterinary community, in particular for treatment of equine wounds (16). Previous work has demonstrated *in vitro* activity against a wide range of equine bacterial isolates (17) with *in vivo* activity also recently reported (18).

Here, we aimed to establish whether manuka honey could inhibit the growth of a range of genetically diverse S. pseudintermedius strains, increase the sensitivity of clinical S. pseudintermedius isolates to a range of antibiotics, and reduce expression of key virulence factors. Activity in these areas would indicate potential for manuka honey in the treatment of difficult-to-treat infections caused by S. pseudintermedius.

## RESULTS

### Genome sequencing showed isolates were genetically diverse and contained novel MLST sequences.

As these isolates have not previously been reported, they were sequenced in order to interrogate their multilocus sequence typing (MLST) and antimicrobial resistance (AMR) profiles and to detect how closely related the isolates were genetically. Sequencing reads and genome assemblies from this study are available from NCBI via the BioProject record PRJNA561036. MLST allele sequences were derived from assemblies and submitted to the Staphylococcus pseudintermedius PubMLST database. New allele sequences were detected for the *pta* locus of isolate C and the *ack* locus of isolate G. A total of 10 novel sequence types (STs) were identified within this data set, with a further 8 STs identified, 4 of which belonged to ST71 (assembly and MLST information can be found in Table S1 in the supplemental material). A summary of the different antibiotic resistance genes present in each isolate can be seen in Table 1.

**TABLE 1:** Summary of the antibiotic resistance genes found by sequencing of each isolate

Isolate identity	Gene(s) by antibiotic class						
	Aminoglyocoside	Beta lactam	Chloramphenicol	Macrolide	Streptothricin	Tetracycline	Trimethoprim
A							
B		*blaI, blaPC1*					
C		*blaI, blaR1, blaZ*				*tet(M)*	
D	*aph(3”)-IIIa, ant(6)-Ia*	*blaPC1, blaR1*				*tet(M)*	
E		*blaI, blaPC1*			*sat4*	*tet(M)*	
F		*blaI, blaR1*					
G							
H	*aph(3”)-IIIa, ant(6)-Ia*	*blaI, blaR1, blaZ*	*catA7*	*erm(B)*	*sat4*	*tet(M)*	
I	*aph(3”)-IIIa, ant(6)-Ia*	*blaI, blaR1*		*erm(B)*	*sat4*		
J							
K		*blaI, blaR1, blaZ*				*tet(M)*	
M		*blaI, blaPC1, blaR1*				*tet(M)*	
N	*aph(3”)-IIIa, ant(6)-Ia*	*blaI, mecA, mecI, mecR1*		*erm(B)*	*sat4*		*dfrG*
O	*aph(3”)-IIIa, ant(6)-Ia*	*blaI, blaZ*	*catA7*	*erm(B)*	*sat4*		
P	*aph(3”)-IIIa, ant(6)-Ia*	*blaI, blaPC1, blaR1, mecA, mecI, mecR1*		*erm(B)*	*sat4*		*dfrG*
Q	*aph(3”)-IIIa*	*blaI, blaR1, mecA, mecI, mecR1*		*erm(B)*	*sat4*	*tet(K)*	*dfrG*
T	*aph(3”)-IIIa, ant(6)-Ia*	*blaI, blaR1, mecA, mecI, mecR1*		*erm(B)*	*sat4*		*dfrG*
X	*aadE*	*blaI, blaPC1, blaR1*		*erm(B)*		*tet(M), tet(O)*	

**FIG 1:**
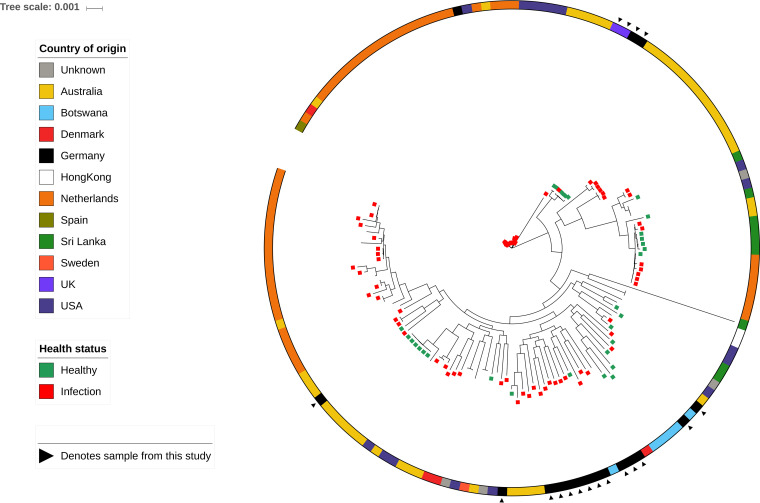
A maximum likelihood phylogeny of 156 Staphylococcus pseudintermedius isolates reconstructed from core SNPs, with removal of detected recombination sites. Samples sequenced in this study are distributed across the tree as noted by the outer black triangles. Colored bars indicate geographical locations of isolation sources. Isolates with metadata related to host health have terminal nodes colored green for healthy or red for confirmed infection.

A core genome SNP phylogeny accounting for the removal of detected recombination and consisting of the isolates documented within this study, along with Staphylococcus pseudintermedius genome assemblies, displayed very little obvious clustering of isolates based on geographical location or the health status of the individual from which they were isolated (Fig. 1).

### Minimum inhibitory/bactericidal concentration testing of honey and antibiotics showed universal activity of honey against the S. pseudintermedius isolates tested.

Previous work by our group indicates that manuka honey has antibacterial activity against *Staphylococcus* sp., including antibiotic-resistant (AR) isolates (11, 13). In this study, all 18 isolates of S. pseudintermedius demonstrated susceptibility to low concentrations of manuka honey (≤12% [wt/vol]), with 9/18 inhibited at 12% (wt/vol) and 9/18 inhibited at 10% (wt/vol) manuka honey. No antimicrobial activity was detected at concentrations of less than 8% (wt/vol) manuka honey. The minimum bactericidal concentration was ≤12% (wt/vol) manuka honey for all isolates.

The sensitivity of the S. pseudintermedius isolates to tetracycline, penicillin, chloramphenicol, gentamicin, and oxacillin was also determined. For tetracycline, penicillin, and gentamicin, more than half of the isolates tested (9, 16, and 11 isolates, respectively) had breakpoints that indicated resistance according to the European Committee on Antimicrobial Susceptibility Testing (EUCAST) guidelines. Only chloramphenicol and oxacillin remained effective against a majority of isolates, with 10 and 14 isolates, respectively, remaining susceptible.

### Assessment of antibiotic interactions showed that honey was able to increase the activity of the majority of antibiotics tested.

There has been significant recent interest in combination therapy, i.e., combinations of antibiotics with other antibiotics, peptides, and plant extracts, to increase antibiotic efficacy (19, 20). Since the isolates within the collection tested here display a high level of resistance to commonly used antibiotic treatments, it was speculated that antibiotic efficacy might be improved if used in combination with sublethal concentrations of manuka honey.

**FIG 2:**
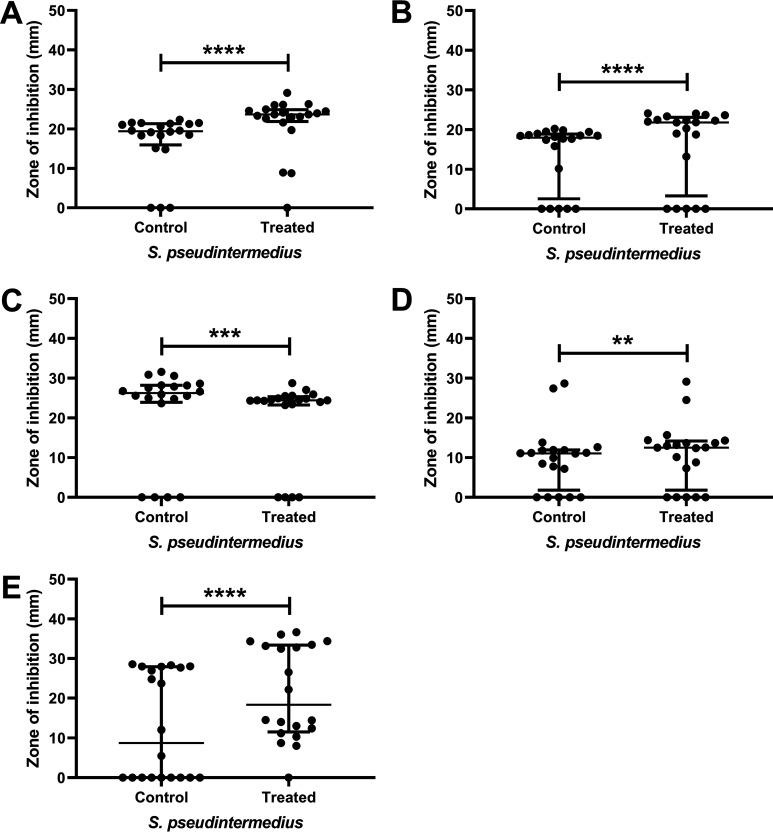
Zone diameters of inhibition. Panels A to E show zone diameters (in mm) with chloramphenicol, gentamicin, oxacillin, penicillin, and tetracycline, respectively, against 18 isolates of S. pseudintermedius in MHB (control) versus a sublethal concentration of manuka honey, 5% (wt/vol) (treated). Those isolates showing significant changes in sensitivity are marked with asterisks (**, *P* ≤ 0.01; ***, *P* ≤ 0.001; ****, *P* ≤ 0.0001). Data points show median values and error bars represent the 95% confidence limit.

Manuka honey increased the sensitivity of a number of isolates to various antibiotics, with small but statistically significant increases in zone size observed for the majority of isolates (Fig. 2, Fig. S1). The exact increase in susceptibility was both strain and antibiotic specific. Full details of the inhibition of each isolate to the five antibiotics with and without honey are shown in Fig. S1. The numbers of isolates which displayed significantly larger zones of inhibition (*P* ≤ 0.05) when treated with sublethal concentrations of honey (5% [wt/vol]) combined with antibiotic compared to antibiotic alone were as follows: tetracycline 89% (16/18), penicillin 56% (10/18), chloramphenicol 83% (15/18), and gentamicin 67% (12/18). In some instances (isolates C, D, E, K, M, and X for tetracycline and isolates O and X for chloramphenicol), the isolates showed complete resistance to the antibiotic in standard medium; however, in the presence of subinhibitory honey, a zone of inhibition was present. When manuka honey was used in combination with oxacillin, none of the isolates displayed increased sensitivity and 33% (6/18) isolates showed significantly decreased sensitivity (Fig. 2, data for individual isolates in Fig. S1).

Generally, there was a good correlation between the AMR phenotype observed in the disc diffusion assays and the genotype of each isolate. There were several instances where discrepancies between genotype and phenotype were detected, but only two are of note. First, no beta lactam-inhibiting genes were detected in isolate G, despite a clear phenotypic resistance to penicillin but not oxacillin. There was a similar observation for isolate X, which was phenotypically resistant to chloramphenicol despite no identifiable resistance gene identified encoding chloramphenicol inactivation enzymes (acquired genes) (Table S1). Thus, in this case, 50S ribosomal subunit mutational effects are the likely source of resistance.

### Characterization of virulence factor activity indicated that manuka honey was able to reduce activity of several key virulence factors.

Alongside its antimicrobial activity, manuka honey has previously been shown to affect protein and gene expression in the closely related species S. aureus (21). It was therefore of interest to determine if manuka honey could elicit phenotypic changes in the virulence profile of S. pseudintermedius isolates. Initially the hemolytic, protease, lipase, and DNase activities of the isolates were assessed without manuka honey, alongside the isolate’s ability to aggregate (a property linked to biofilm formation).

**FIG 3:**
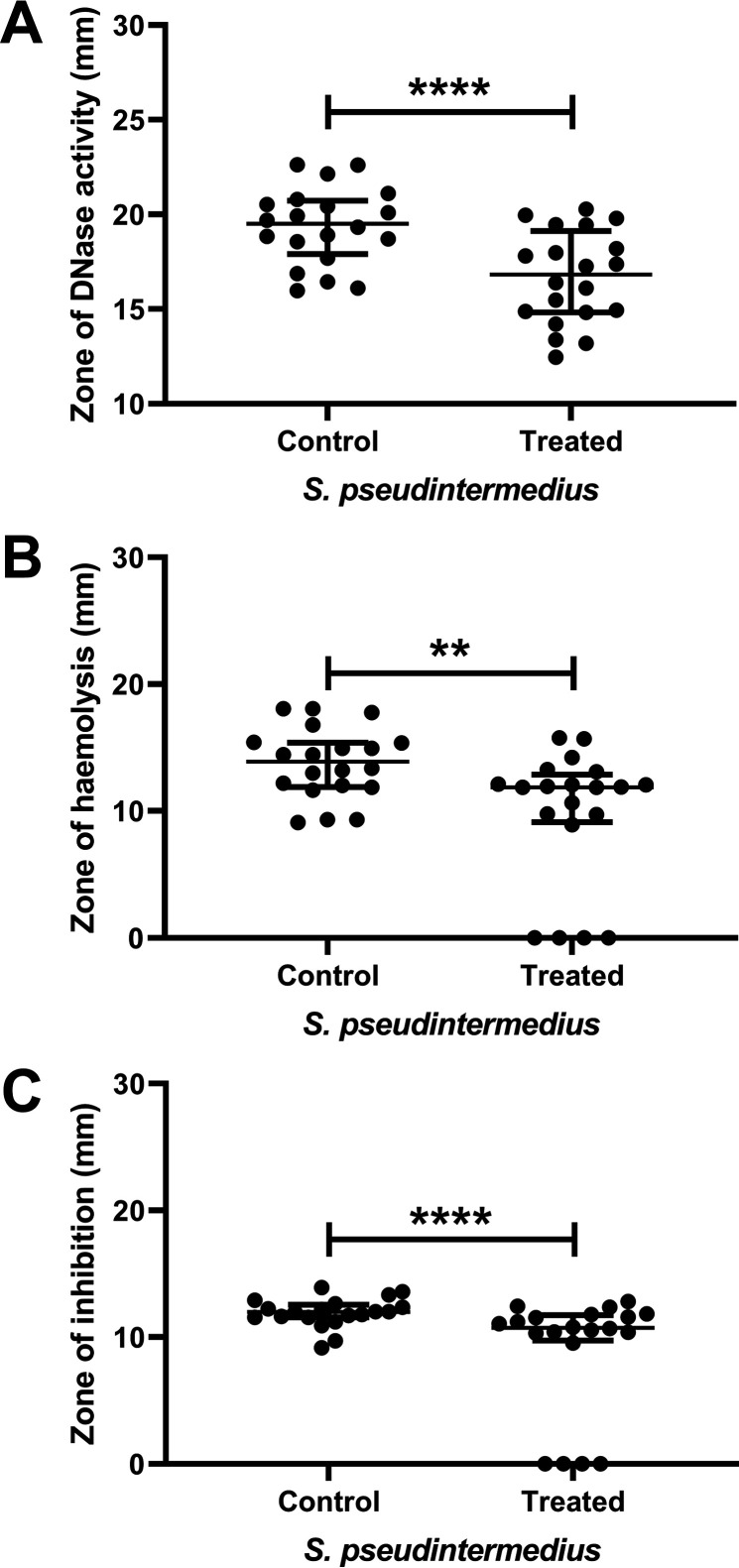
Manuka honey is able to inhibit virulence factor production by S. pseudintermedius. DNase (A), hemolytic (B), and proteolytic (C) activities of the 18 S. pseudintermedius isolates in the absence (control) or presence (treated) of a sublethal (5% [wt/vol]) concentration of manuka honey. Those isolates showing significant changes in sensitivity are marked with asterisks (**, *P* ≤ 0.01; ****, *P* ≤ 0.0001). Data points show median values and error bars represent the 95% confidence limit.

All 18 isolates showed hemolytic, proteolytic, DNase, and aggregation activities. None of the isolates demonstrated lipase or lecithinase capabilities (data not shown). The activity was then reassessed using a sublethal concentration (5% [wt/vol]) of manuka honey. As with the antibiotic activity (Fig. 2), the presence of subinhibitory concentrations of honey were able to elicit alterations in the virulence activity of the S. pseudintermedius isolates. As with the alterations in antibiotic susceptibility, alterations in virulence activity appeared to be highly isolate specific. In most of the isolates the observed effects were subtle, although statistically significant. However, four of the strains showed a complete reduction of hemolysis (strains G and H) or proteolysis (strains B and C) activity. Two further strains (D and F) showed complete reduction of both hemolysis and proteolysis. The numbers of isolates which had significantly changed (*P* ≤ 0.05) hemolysis, proteolysis, and DNase activities when grown in a sublethal concentration of honey (5% [wt/vol]) compared to control cells were hemolytic 56% (10/18), protease 50% (9/18), and DNase 72% (13/18) (Fig. 3, Fig. S2).

### Subinhibitory concentrations of manuka honey inhibited aggregation and reduced mature biofilm biomass of S. pseudintermedius suspensions.

**FIG 4:**
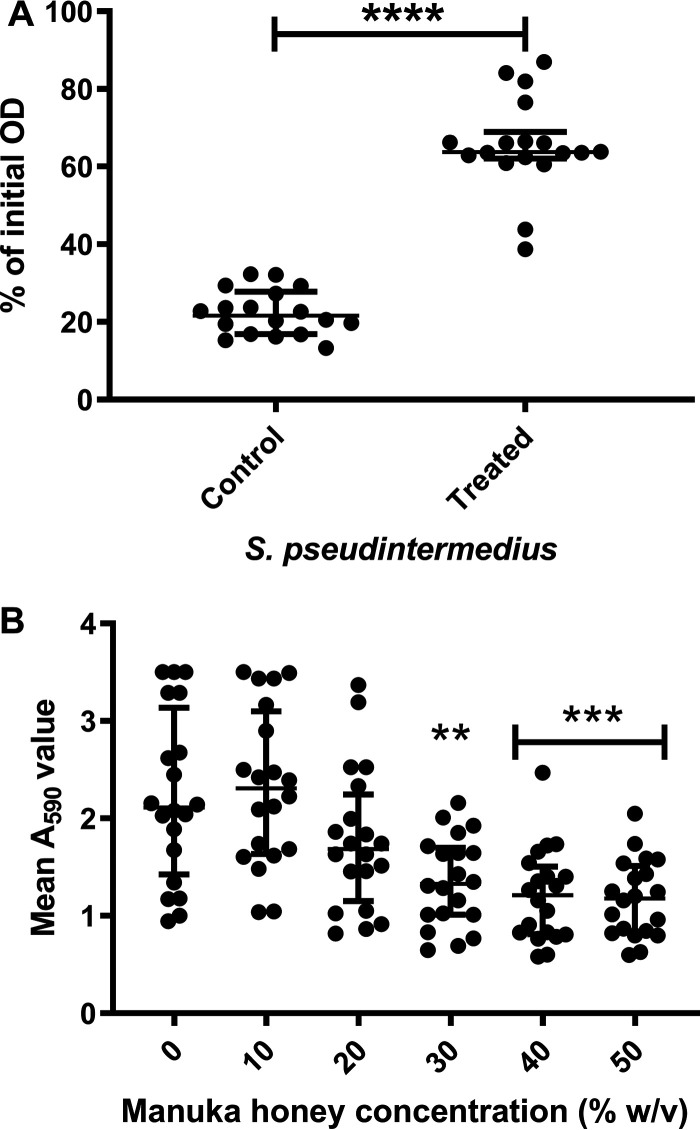
The ability of S. pseudintermedius isolates to aggregate is reduced in the presence of subinhibitory 5% (wt/vol) manuka honey. (A) Effect of sublethal (5% [wt/vol]) manuka honey on 18 S. pseudintermedius isolates. Isolates showed a significant decrease (*P* ≤ 0.001) in their ability to aggregate when treated with 5% (wt/vol) manuka honey. (B) Effect of increasing concentrations of manuka honey on preformed 24-h biofilms with a significant (**, *P* ≤ 0.01; ***, *P* = 0.001) reduction in biofilm seen at concentrations of ≥30% (wt/vol). Data points indicate median values and error bars represent the 95% confidence limit.

All 18 isolates displayed the ability to agglutinate. After 24 h of incubation, control isolates had a mean optical density at 600 nm (OD_600_) of 25% of the 0-h value, indicating that bacterial cells had agglutinated, leading to a decreased OD_600_ (Fig. 4A). In contrast, 15 of the isolates displayed a significantly reduced (*P* ≤ 0.001) ability to agglutinate after incubation with a sublethal (5% [wt/vol]) concentration of honey, with a mean optical density of 65% of the 0-h value (Fig. 4A). Total viable cell counts after 24 h of incubation with and without manuka honey highlighted that the increased optical density was not linked to an increase in cell numbers, with total viable counts indicating that ∼4 × 10^7^ CFU/ml viable cells remained in each vessel following incubation. With the exception of isolates B and H, which were moderate biofilm formers, and isolate O, which was a weak biofilm former, all the isolates were strong biofilm formers as defined by Stepanovic et al. (22).

As expected, the concentrations of manuka honey needed to disrupt established (24 h) biofilm were higher than the MIC (Fig. 4B). There was a reduction in biomass at concentrations of ≥20% (wt/vol) manuka honey, with reductions becoming significant (*P* ≤ 0.01) at concentrations of ≥30%.

## DISCUSSION

The increase in S. pseudintermedius antibiotic resistance is of concern to both clinicians and veterinarians (23, 24). New strategies for infection control are required and a One Health approach must be utilized during discovery to ensure that therapeutics can be used harmoniously across medical disciplines. Clinicians and veterinarians have previously been criticized for excessive use of antibiotics (25), and following the publication of the “Review on Antimicrobial Resistance” (chaired by Jim O'Neill) (26) there has been a drive to improve collaboration between medical, veterinary, and agricultural disciplines (27) in addressing the issue of AMR. The report by O'Neill (26) also highlighted that the development of novel therapeutics to enhance the activity of, or replace, antibiotic treatments was an essential strategy to preserve our ability to effectively treat infectious diseases.

Zoonotic pathogens are of concern, since inappropriate treatment and resolution of animal infections has the potential to increase disease severity and recalcitrance to treatment of any subsequent human infection. Other members of the *Staphylococcus* genus, such as S. aureus and S. epidermidis, are persistent colonizers of the human body, causing significant morbidity and mortality, and display high levels of AMR. S. pseudintermedius has also been shown to colonize healthy humans (28) and appears to have similar disease progression and resistance patterns to other members of the genus (23, 24, 29).

The S. pseudintermedius strains in this study showed a wide range of genetic diversity (Fig. 1), including the presence of several novel MLST combinations. The genetic diversity of these samples allowed an estimation of how effective manuka honey might be for treatment of S. pseudintermedius infections, as multiple strains of S. pseudintermedius with varied antibiotic resistance profiles can cocolonize at an infection site (30). The majority of isolates also contained genes known to confer antibiotic resistance, with many isolates also showing phenotypic resistance to one or more of the antibiotics tested. Only in a couple of instances was there a discrepancy between the phenotypic resistance patterns and the presence of acquired AMR genes. However, since the genomes presented within this paper are not closed, it is possible that genes are present in regions of the genome currently unassembled. Further, the presence of point mutations giving rise to resistance were not assessed and could thus offer alternative resistance strategies.

The results presented here provide evidence that low concentrations of manuka honey can inhibit the growth of clinical isolates of S. pseudintermedius, with a bactericidal mode of action. All isolates displayed sensitivity to manuka honey and had a range of sensitivities to the conventional antibiotics tested. This is in line with previous studies, which have demonstrated that manuka honey can inhibit S. aureus, MRSA, and vancomycin-intermediate S. aureus (VISA) at low concentrations (31–33). The only study looking at the ability of manuka honey to inhibit S. pseudintermedius was by Uri et al. (34), and that study showed a low level of antibacterial activity. However, this was likely due to issues with the homogeneity of the honey solution used in the study, as highlighted by the authors of that paper, and not a true reflection of manuka honey efficacy, particularly given the many *in vitro* and *in vivo* studies showing efficacy against other bacterial species (11, 13, 21).

The results of the antibiotic susceptibility testing presented here reflect the general trend of increased antimicrobial resistance seen globally, with 88% of the isolates tested displaying resistance to two or more of the antibiotics (Fig. 2). The levels of resistance for gentamicin and chloramphenicol presented here are broadly similar to findings from earlier studies, where S. pseudintermedius resistance to chloramphenicol and gentamicin was ∼75 and 55%, respectively, whereas the levels of resistance for tetracycline were lower in this study (at ∼50%) than those previously reported (94.2%) (35, 36). There were only four MRSP isolates within our collection, a lower incidence of resistance than previously reported (35, 36).

In addition to obtaining the sensitivity profiles of these bacteria, and in view of the growing interest in the use of topical and combination therapies for staphylococcal infections (37), the ability of manuka honey to enhance antibiotic activity was tested. Our results highlight how sublethal concentrations of honey are capable of significantly improving (*P* < 0.05) the activity of antibiotics from a variety of classes, for most isolates tested (Fig. 2). The exception to this was oxacillin, for which combination with honey showed no improvement in activity against any of the MRSP isolates. Importantly, in six of the methicillin-susceptible S. pseudintermedius (MSSP) isolates, manuka honey was associated with a significant decrease in the efficacy of oxacillin, although the isolates did remain sensitive to oxacillin according to the breakpoints. This was an unexpected finding, as previous studies have identified synergy between honey and oxacillin when tested against MRSA, as well as reporting a possible mechanism of action for this synergy through the MecR1 pathway (32, 38). It is interesting to note that synergy has been observed for MRSA with the *mecA* mechanism of resistance previously, and the four MRSPs here all have resistance mediated by *mecA* (Table S1), the same as seen in the MRSA, so a similar response would have been expected.

This difference in response could be investigated further in future work. That the isolates with the altered response are phenotypically methicillin sensitive, and therefore unlikely to contain a functioning *mecR1* gene, highlights why the response is different from that previously observed. The *mecR1* gene has not been shown to be common within S. pseudintermedius isolates, with a recent study only detecting it in 7 of 17 isolates tested (39). It has also been observed that some MRSP isolates do not contain *mecR1* (40), again showing that even where isolates do have phenotypic resistance to penicillin, honey may not be able to act on them via the previously described MecR1 pathway. The observed reduction in sensitivity to oxacillin upon concurrent exposure to sublethal concentrations of manuka honey is an important observation for medical practice, where oxacillin is a commonly used treatment, as it suggests that using manuka honey in combination with the other antibiotics tested here would be more effective than oxacillin as part of a potential combination therapy. Our observation also highlights the need, previously stressed within the O’Neill report (26), for novel bedside diagnostic tools in order to better determine suitable treatments for patients presenting with infection. If clinicians are able to rapidly distinguish between opportunistic infections caused by S. aureus and S. pseudintermedius, then suitable combination therapies can be selected, improving clinical outcome and reducing the opportunity for microbial persistence. Within veterinary practice, where S. pseudintermedius infection is more commonly encountered, oxacillin is not considered for first line treatment of skin infections (41) and thus our observation is of less significance. It should also be noted that our data did not show decreased susceptibility to penicillin, indicating that manuka honey/oxacillin combination therapies may not be suitable for treatment of S. pseudintermedius, though this is unlikely to be the case for all penicillin. Investigation into the mechanism by which honey is inhibiting S. pseudintermedius infection might shed light onto why there is this discrepancy between S. pseudintermedius and S. aureus when treated with oxacillin and honey; genetic variation between the two species might account for the reduced efficacy of the combination in this case. Despite this, and given the high levels of antibiotic resistance increasingly reported, the results presented here show there is real potential for honey to be used alone or as a topical adjuvant to certain antibiotic treatments to help improve antibiotic efficacy against S. pseudintermedius infections.

Alongside investigation of synergy, antivirulence compounds are of great interest as they reduce pathogenicity while leaving bacterial growth unaffected and desirable host microbes unharmed (42). As S. pseudintermedius strains are known to produce a range of virulence factors that enhance their ability to cause severe disease in both animals and humans (23, 43), the effect of sublethal concentrations of manuka honey on the activity of virulence factors in S. pseudintermedius was tested. Investigating the effect of sublethal concentrations of honey on virulence provided novel evidence that honey can significantly reduce virulence factor activity in some S. pseudintermedius isolates (Fig. 3). In infection, the ability to produce an extensive array of virulence factors allows bacteria to break down host tissue, evade the immune system, and acquire host nutrients (5, 44). The isolates in this study all displayed various levels of β hemolytic, protease, and DNase activity, as well as the ability to auto-agglutinate. They were all negative for lipase and lecithinase activity, correlating well with previous findings charting the virulence profiles of S. pseudintermedius isolates from humans and dogs (45). The data presented here establishes that sublethal concentrations of honey significantly reduced hemolysin activity in half the S. pseudintermedius isolates tested, indicating a strain-specific mode of action. As there was a significant increase in hemolytic activity of one isolate, further work would be needed before this could be considered of interest in a clinical setting. To date, there is little evidence of the role of β-hemolysin in S. pseudintermedius pathogenicity. In S. aureus, β-hemolysin production promotes efficient skin colonization (46). It is also thought to cause host cell cytotoxicity, act as a biofilm ligase, and confer a selective advantage to those strains which produce it (47). It is thus logical to suggest the ability of honey to reduce β hemolytic activity would thereby reduce the capability of S. pseudintermedius to colonize host cells and cause cytotoxicity and reduce zoonotic transmission.

Similarly, the results presented here show that sublethal doses of manuka honey significantly reduce the ability of half the S. pseudintermedius strains to produce protease and did not increase protease activity in any of the other strains. As the ability of staphylococci to produce proteases has been linked to their ability to evade host immunity, as well as cleaving host proteins and contributing to bacterial dissemination within the host (48, 49), reduction in this activity could be clinically relevant. Previously published work showed that protease null mutants of S. aureus have decreased virulence and reduced dissemination and invasion *in vivo* (50). S. pseudintermedius produces a similar range of proteases and, if they perform an analogous role to those seen in S. aureus, then honey could have an important function in decreasing the severity of localized infection and reducing the spread of S. pseudintermedius within the host.

Linked to infection duration and antibiotic tolerance is DNase activity, which has been reported to aid in the formation of bacteria aggregates, maintenance of biofilm (51), and degradation of neutrophil extracellular traps (52). These actions all assist the bacteria in evading the immune system. The reduction of DNase activity seen in over half the S. pseudintermedius tested (13/18) again suggests a role for manuka honey in reducing bacterial virulence and supporting host function in infection. It is possible that the significant decrease in DNase activity after treatment with sub-MIC honey in this study could be related to the significant reduction in aggregation also observed. The ability to agglutinate has been linked to the ability of isolates to form biofilms, an important mechanism of persistence (53).

Aggregation is known to be partially dependent on components displayed on the cell surface (54), but the role of DNase in *Staphylococcus* aggregation is less well understood. Some studies observed increased expression of DNase from planktonic cells (55), while others describe increased expression of DNase from sessile cells (56). It has been shown that the presence of extracellular DNA (eDNA) improves the ability of bacteria to aggregate and adhere to surfaces (57), therefore a reduction in DNase expression would be expected to lead to increased aggregation. As exposure to sublethal concentrations of manuka honey led to a reduction in both DNase activity and bacterial aggregation, there could well be additional factors at work here. Further work to elucidate the mechanisms which cause these effects needs to be undertaken.

The ability to form, and persist within, biofilms is a known *Staphylococcus* virulence trait which decreases the susceptibility of *in vivo* populations to antimicrobial treatment and provides a reservoir for systemic infection (58). Significant effort has been dedicated to removing and inactivating the biofilms of S. aureus and S. epidermidis, with manuka honey showing significant promise in both species (11, 59, 60). The data presented here suggests that S. pseudintermedius isolates are likely no exception to this trend, with all the isolates within our collection showing a statistically significant reduction in mature biofilm biomass at manuka honey concentrations of ≥30% (wt/vol). This suggests that manuka honey is able to disperse preformed biofilms and that individual individual shed from the biofilms will rapidly succumb to the antimicrobial effects that manuka honey showed against planktonic populations.

The data presented show that *in vitro* manuka honey is able to reduce the activity of the virulence factors tested; however, caution should be used when interpreting the data as the changes seen *in vitro* might not be replicated *in vivo*. Although the activity assays used within this study allow investigators to measure differences in activity of the supernatant, this technique has limitations as it does not distinguish between an alteration in activity due to changes in protein efficiency versus effects produced by changes in gene expression and/or protein production. In order to provide more information about the exact mechanism of virulence factor modulation by manuka honey, the techniques used here must be combined with an analysis of gene presence (whole-genome sequencing [WGS]), gene expression (quantitative PCR [qPCR] and RNAseq), and protein expression (2D gel electrophoresis and mass spectrometry). These additional analyses, while outside the scope of the current work, would be of significant interest to help elucidate manuka honey’s mechanism of activity against S. pseudintermedius. Further *in vivo* testing will be required to ensure that significant reductions in activity highlighted here translate to a biological difference in the virulence of the bacteria within animals and humans.

It is clear from the results presented here that manuka honey alone can effectively inhibit antibiotic-sensitive and resistant S. pseudintermedius at low concentrations and, when combined with selected antibiotics, can increase their efficacy. It is also apparent that the addition of sublethal doses of manuka honey *to* S. pseudintermedius has multiple effects on the virulence activity of those isolates, with over half seeing a significant reduction in virulence activity *in vitro*. S. pseudintermedius is recognized as a major cause of postoperative infections in small animals and, more recently, as a colonizer and opportunistic pathogen of humans (6). Our data indicate that use of manuka honey in clinical treatment against S. pseudintermedius infections could be possible and that manuka honey could potentially be used as an antibiotic adjuvant for difficult-to-treat multiantibiotic resistant strains. Further investigation via broth microdilution on the effect of manuka honey on antibiotic sensitivity would help determine which interactions are antagonistic, indifferent, or synergistic. In addition, there is strong evidence that manuka honey could function as a topical antivirulence treatment to help reduce the severity of S. pseudintermedius infections and limit further colonization. Investigations into the mechanisms by which manuka honey is eliciting these effects needs to be undertaken to determine the mechanistic processes and strain-specific effects observed.

## MATERIALS AND METHODS

### Strains and culture conditions.

Eighteen S. pseudintermedius isolates from dogs were used throughout this study. All were provided by Nottingham Veterinary School, UK (18 isolates). These cultures were stored at −80°C on cryobeads and revived onto Muller-Hinton agar (MHA) (Oxoid, UK) at 37°C before testing. Throughout the experiments, Mueller-Hinton broth (MHB) was used where liquid medium was required.

### Antimicrobial agents.

Sterile medical-grade manuka honey (Derma Sciences, Medihoney) was used throughout and was a gift from Derma Sciences, Europe. Antimicrobial susceptibility testing discs of penicillin (1 unit), tetracycline (30 μg), chloramphenicol (30 μg), gentamicin (10 μg), and oxacillin (1 μg) were purchased from Oxoid (Hampshire, UK). These concentrations were chosen to match break points in the EUCAST breakpoint tables.

### DNA extraction/sequencing.

DNA was extracted from cultures using the Qiagen DNAeasy kit according to the manufacturer’s guidelines prior to quantifying yield and quality using spectrophotometric and fluorometric methods. DNA libraries for sequencing were constructed using the Illumina Nextera XT kit and sequenced on an Illumina MiSeq platform using a V3 reagent kit and 2 × 300 bp reads.

### Genome assembly and analysis.

Raw sequence reads were passed through cutadapt (v2.0) (61) for the removal of nextera sequence adapters only prior to genome assembly using SPAdes (v3.12.0) in careful mode with read error correction and auto k-mer detection (62). Quast (v5.0.0) (63) was used to assess the quality of the genome assemblies. MLST allele sequences, IDs, and isolate STs were queried against the genome assemblies using the MLST tool (v2.16) (currently unpublished, T. Seemann, mlst [Github, https://github.com/tseemann/mlst]). A core SNP phylogeny was reconstructed using the isolates from this study along with 137 S. pseudintermedius genome assemblies obtained from NCBI and using variant sites called by SNIPPY (v4.3.2) (currently unpublished, T. Seemann, SNIPPY [Github, https://github.com/tseemann/SNIPPY]). Recombination was accounted for with the use of Gubbins (v 2.3.1) (64) prior to inference of a maximum-likelihood phylogenetic tree using FastTree (v 2.1.10), applying the generalized time-reversible model (gtr) (65). A final tree was annotated with the use of iTOL (66). The presence of antimicrobial resistance genes was determined using ABRICATE (v0.8.11) (T. Seemann, Abricate [Github, https://github.com/tseemann/abricate]). A threshold of >90% sequence identity was used to determine a good match between the database genes and hits within the genome sequences.

### Minimum inhibitory/bactericidal concentration testing.

MICs of manuka honey antimicrobial susceptibility were assessed using an adapted European Committee on Antimicrobial Susceptibility Testing methodology. Briefly, mid-log-phase cells were diluted to 0.5 McFarland standard diluted 1 in 100 before it was mixed 1:1 with MHB to a final volume of 200 μl (creating a cell density of ∼5 × 10^5^ CFU/ml) with the manuka honey set out as 2% (wt/vol) increments, diluted in MHB. The isolates were then grown for a period of 16 to 20 h at 37°C before measurement of optical density at a wavelength of 600 nm (OD_600_). The MIC was considered to be the lowest concentration which inhibited cell growth (defined as blank-corrected OD_600_ measurements equaling ≤0). The minimum bactericidal concentration (MBC) was determined by plating 10 μl of cells onto MHA from all wells showing no growth at 18 h and incubating for a further 18 h at 37°C. The MBC was defined as the lowest concentration that reduced the viability of the initial bacterial inoculum by ≥99.9%. Positive-control wells contained growth medium and bacterial cells with no antibiotic or honey, negative-control wells (used for blank correction) contained growth medium and no cells.

### Antibiotic disc susceptibility.

Susceptibility of isolates to antibiotics alone and in combination with sublethal concentrations of manuka honey was determined using disc diffusion on MHA. Briefly, the cells were diluted to 0.5 McFarland standard and then, using a sterile cotton swab, a lawn plate was created and antibiotic discs were immediately applied using sterile forceps. The plates were incubated for 16 to 20 h at 37°C before the diameter of the zones of inhibition was measured. Zone sizes were interpreted using EUCAST Clinical Breakpoint Tables v. 9.0; where S. pseudintermedius breakpoints were unavailable, those for S. aureus were used. To screen for potential synergy between antibiotics and honey, the disc diffusion assay was repeated with MHA supplemented poststerilization with a sublethal dose of 5% (wt/vol) manuka honey.

### Virulence testing.

All isolates were screened for their ability to produce the virulence factors hemolysin, protease, lipase, lecithinase, and DNase. Agar plates were prepared by supplementing MHA with either 5% (vol/vol) sheep blood, 5% (vol/vol) skim milk powder, or 5% (vol/vol) egg yolk (Sigma, UK). Supplements were added after the medium had been autoclaved and were mixed before pouring into plates. DNase agar was purchased directly from Sigma and prepared following the manufacturer’s guidelines. Prior to use, the plates were equilibrated to room temperature and 5-mm diameter wells were cut in the agar surface using a sterile cork borer. Overnight cultures were diluted to a density of OD_600_ 0.8 to 1.0 (equivalent to a cell density of ∼1 × 10^8^ CFU/ml) and pelleted before resuspension in 0.4% MHA, with or without 5% (wt/vol) honey. A volume of 100 μl of this adjusted cell suspension was added to the plate wells and the plates cooled quickly to allow the soft agar to rapidly set and form a plug within the well. The plates were then incubated for 24 h at 37°C and a zone of activity around bacterial growth was recorded as a positive result for each virulence factor.

### Auto-aggregation.

To assess aggregation, overnight cultures were adjusted to OD_600_ 0.9 and then 1 ml was transferred to a 1.5-ml Eppendorf tube, centrifuged at 14,000 × *g* for 5 min, and the pellet resuspended in 1 ml of phosphate-buffered saline (PBS) (Oxoid, UK) or PBS + honey (5% [wt/vol]). The inoculum was then transferred to microcuvettes and the OD_600_ measured at 0 and 24 h with static incubation at room temperature between the measurements. Bacterial cells that strongly agglutinate do not remain in the aqueous phase, leading to a decrease in the OD_600_, whereas cells prevented from agglutinating show an OD_600_ that remains relatively stable. The percentage change of OD_600_ was calculated after 24 h. Viable cell counts were taken using the Miles Misra method before and after incubation to confirm that any differences in auto-aggregation were not due to cell death or proliferation.

### Biofilm assessment.

To determine the effect of manuka honey on mature biofilm, S. pseudintermedius strains were diluted as described for MIC testing and diluted 1:1 with MHB to a final volume of 200 μl before incubating for 24 h at 37°C to allow biofilm formation. Following incubation, the supernatant was removed carefully from the wells, so as not to disturb the biofilm, and washed once with sterile PBS. Manuka honey was diluted to concentrations of 10% to 50% (wt/vol) (in increments of 10%) in MHB. A volume of 200 μl of these solutions was added to the biofilms before incubating for a further 24 h at 37°C. Following incubation, supernatant was removed and wells gently rinsed twice with PBS before fixing remaining biofilms with methanol for 15 min and staining with crystal violet (1% [vol/vol] diluted in distilled water) for 15 min. Excess stain was rinsed until water ran clear and wells were dried before adding 7% acetic acid (diluted in distilled water) for 15 min to dissolve the bound crystal violet. Absorbance was measured at a wavelength of 590 nm.

The ability of the S. pseudintermedius strains to attach to surfaces and form biofilm was performed following the method and classification proposed by Stepanovic et al. (22). Biofilm cultures were established, fixed, and stained as described above and then classified as weakly, moderately, or strongly adherent, based upon the following formula: OD_C_ < OD ≤ 2× OD_C_ = weak adherence; 2× OD_C_ < OD ≤ 4× OD_C_ = moderate adherence; and 4× OD_C_ < OD = strong adherence. OD_C_ was defined as three standard deviations above the mean OD of the negative (medium only) control.

### Statistical analysis.

All tests were performed in triplicate; as data were nonparametric (determined using a Shapiro-Wilk test), they were analyzed using the Mann-Whitney method (for auto-aggregation) and the Kruskal Wallis method (for all other experiments) (GraphPad Prism 8). Unless otherwise stated, all bars in figures show median values and error bars represent a 95% confidence limit.

### Data availability.

Sequencing reads and genome assemblies from this study are available from NCBI via the BioProject record PRJNA561036.

Accession numbers are as follows: isolate A (SAMN12347716, SRR9998529), isolate B (SAMN12347717, SRR9998521, SRR9998530), isolate C (SAMN12347718, SRR9998528, SRR9998519), isolate D (SAMN12347719, SRR9998518), isolate E (SAMN12347720, SRR9998522), isolate F (SAMN12347721, SRR9998525), isolate G (SAMN12347722, SRR9998517), isolate H (SAMN12347723, SRR9998524), isolate I (SAMN12347724, SRR9998523), isolate J (SAMN12347725, SRR9998520), isolate K (SAMN12347726, SRR9998533), isolate M (SAMN12347727, SRR9998534), isolate N (SAMN12347728, SRR9998535), isolate O (SAMN12347729, SRR9998536), isolate P (SAMN12347730, SRR9998526, SRR9998527), isolate Q (SAMN12347731, SRR9998531), isolate T (SAMN12347732, SRR9998516), isolate X (SAMN12347733, SRR9998532).

## Supplementary Material

Supplemental file 1
