# Supplementary material for: Antibacterial and Antivirulence Activity of Manuka Honey against Genetically Diverse Staphylococcus pseudintermedius Strains
Source: Appl Environ Microbiol. 2020 Oct 1;86(20):e01768-20. doi: 10.1128/AEM.01768-20 (PMC7531947; doi:10.1128/AEM.01768-20)
Supplement: Supplemental file 1 [file AEM.01768-20-s0001.pdf]

Supplementary Table 1

Isolate ID	SEQUENCE	START	END	STRAND	GENE	COVERAGE	%COVERAGE	%IDENTITY	DATABASE	ACCESSION	PRODUCT	RESISTANCE
B	NODE_48_length_17889_cov_4.750084	2531	2904	+	blaI_of_Z	1-381/381	98.16	94.49	ncbi	NG_047499.1	penicillinase repressor BlaI	BETA-LACTAM
B	NODE_48_length_17889_cov_4.750084	1	835	-	blaPC1	1-835/846	98.7	99.28	ncbi	NG_052065.1	BlaZ family penicillin-hydrolyzing class A beta-lactamase PC1	BETA-LACTAM
C	NODE_7_length_57436_cov_2.482106	44275	44655	-	blaI_of_Z	1-381/381	100	95.8	ncbi	NG_047499.1	penicillinase repressor BlaI	BETA-LACTAM
C	NODE_7_length_57436_cov_2.482106	44645	46385	-	blaR1	1-1758/1758	99.03	96.25	ncbi	NG_047539.1	beta-lactam sensor/signal transducer BlaR1	BETA-LACTAM
C	NODE_7_length_57436_cov_2.482106	46494	47324	+	blaZ	6-846/846	98.23	98.69	ncbi	NG_055999.1	penicillin-hydrolyzing class A beta-lactamase BlaZ	BETA-LACTAM
C	NODE_45_length_14323_cov_14.356650	914	1294	+	blaI_of_Z	1-381/381	100	100	ncbi	NG_047499.1	penicillinase repressor BlaI	BETA-LACTAM
C	NODE_25_length_29038_cov_3.022967	12732	14648	-	tet(M)	1-1920/1920	99.84	99.69	ncbi	NG_048252.1	tetracycline resistance ribosomal protection protein Tet(M)	TETRACYCLINE
D	NODE_12_length_60787_cov_18.093043	59288	60133	-	blaPC1	1-846/846	100	99.41	ncbi	NG_052065.1	BlaZ family penicillin-hydrolyzing class A beta-lactamase PC1	BETA-LACTAM
D	NODE_45_length_14323_cov_14.356650	1	924	+	blaR1	835-1758/1758	52.56	99.89	ncbi	NG_047539.1	beta-lactam sensor/signal transducer BlaR1	BETA-LACTAM
D	NODE_23_length_39788_cov_15.775623	14752	16671	+	tet(M)	1-1920/1920	100	100	ncbi	NG_048252.1	tetracycline resistance ribosomal protection protein Tet(M)	TETRACYCLINE
E	NODE_2_length_293846_cov_18.676841	72746	73540	+	aph(3')-IIIa	1-795/795	100	100	ncbi	NG_047418.1	aminoglycoside O-phosphotransferase APH(3')-IIIa	AMIKACIN/ KANAMYCIN
E	NODE_14_length_57344_cov_25.165003	55814	56194	-	blaI_of_Z	1-381/381	100	95.8	ncbi	NG_047499.1	penicillinase repressor BlaI	BETA-LACTAM
E	NODE_24_length_27901_cov_29.657377	903	1748	+	blaPC1	1-846/846	100	99.29	ncbi	NG_052065.1	BlaZ family penicillin-hydrolyzing class A beta-lactamase PC1	BETA-LACTAM
E	NODE_24_length_27901_cov_29.657377	1	796	-	blaR1	1-796/1758	45.28	99.87	ncbi	NG_047536.1	beta-lactam sensor/signal transducer BlaR1	BETA-LACTAM
E	NODE_14_length_57344_cov_25.165003	56184	57244	-	blaR1	698-1758/1758	60.35	97.55	ncbi	NG_047536.1	beta-lactam sensor/signal transducer BlaR1	BETA-LACTAM
E	NODE_2_length_293846_cov_18.676841	71206	72114	+	ant(6)-Ia	1-909/909	100	100	ncbi	NG_047393.1	aminoglycoside nucleotidyltransferase ANT(6)-Ia	STREPTOMYCIN
E	NODE_2_length_293846_cov_18.676841	72111	72653	+	sat4	1-543/543	100	100	ncbi	NG_048072.1	streptothricin N-acetyltransferase Sat4	STREPTOTHRICIN
E	NODE_1_length_405048_cov_24.241768	285597	287516	+	tet(M)	1-1920/1920	100	100	ncbi	NG_048250.1	tetracycline resistance ribosomal protection protein Tet(M)	TETRACYCLINE
F	NODE_24_length_16549_cov_33.756059	15050	15430	-	blaI_of_Z	1-381/381	100	95.8	ncbi	NG_047499.1	penicillinase repressor BlaI	BETA-LACTAM
F	NODE_24_length_16549_cov_33.756059	15420	16549	-	blaR1	629-1758/1758	64.28	95.66	ncbi	NG_047536.1	beta-lactam sensor/signal transducer BlaR1	BETA-LACTAM
H	NODE_32_length_7578_cov_21.847806	3184	3978	-	aph(3')-IIIa	1-795/795	100	100	ncbi	NG_047418.1	aminoglycoside O-phosphotransferase APH(3')-IIIa	AMIKACIN/ KANAMYCIN
H	NODE_22_length_27273_cov_19.866868	22609	22989	-	blaI_of_Z	1-381/381	100	95.01	ncbi	NG_047499.1	penicillinase repressor BlaI	BETA-LACTAM
H	NODE_4_length_160575_cov_28.113202	158473	158853	-	blaI_of_Z	1-381/381	100	100	ncbi	NG_047499.1	penicillinase repressor BlaI	BETA-LACTAM
H	NODE_4_length_160575_cov_28.113202	158843	160575	-	blaR1	26-1758/1758	98.58	99.94	ncbi	NG_047539.1	beta-lactam sensor/signal transducer BlaR1	BETA-LACTAM

H	NODE_22_length_27273_cov_19.866868	22979	24736	-	blaR1	1-1758/1758	100	92.83	ncbi	NG_047539.1	beta-lactam sensor/signal transducer BlaR1	BETA-LACTAM
H	NODE_22_length_27273_cov_19.866868	24843	25688	+	blaZ	1-846/846	100	98.34	ncbi	NG_055999.1	penicillin-hydrolyzing class A beta-lactamase BlaZ	BETA-LACTAM
H	NODE_33_length_7545_cov_23.303047	4340	4987	+	catA7	1-648/648	100	100	ncbi	NG_047568.1	type A-7 chloramphenicol O-acetyltransferase	CHLORAMPHENICOL
H	NODE_33_length_7545_cov_23.303047	199	936	-	erm(B)	1-738/738	100	100	ncbi	NG_047794.1	23S rRNA (adenine(2058)-N(6))-methyltransferase Erm(B)	MACROLIDE
H	NODE_32_length_7578_cov_21.847806	4610	5518	-	ant(6)-Ia	1-909/909	100	100	ncbi	NG_047393.1	aminoglycoside nucleotidyltransferase ANT(6)-Ia	STREPTOMYCIN
H	NODE_32_length_7578_cov_21.847806	4071	4613	-	sat4	1-543/543	100	100	ncbi	NG_048072.1	streptothricin N-acetyltransferase Sat4	STREPTOTHRICIN
H	NODE_4_length_160575_cov_28.113202	106079	107998	-	tet(M)	1-1920/1920	100	99.95	ncbi	NG_048252.1	tetracycline resistance ribosomal protection protein Tet(M)	TETRACYCLINE
I	NODE_63_length_7203_cov_21.646269	3667	4461	+	aph(3')-IIIa	1-795/795	100	100	ncbi	NG_047418.1	aminoglycoside O-phosphotransferase APH(3')-IIIa	AMIKACIN/KANAMYCIN
I	NODE_39_length_18646_cov_51.382742	1681	2061	+	blaI_of_Z	1-381/381	100	95.8	ncbi	NG_047499.1	penicillinase repressor BlaI	BETA-LACTAM
I	NODE_39_length_18646_cov_51.382742	1	1691	+	blaR1	68-1758/1758	96.19	98.4	ncbi	NG_047536.1	beta-lactam sensor/signal transducer BlaR1	BETA-LACTAM
I	NODE_59_length_10667_cov_38.954649	9633	10370	+	erm(B)	1-738/738	100	100	ncbi	NG_047802.1	23S rRNA (adenine(2058)-N(6))-methyltransferase Erm(B)	MACROLIDE
I	NODE_63_length_7203_cov_21.646269	2127	3035	+	ant(6)-Ia	1-909/909	100	100	ncbi	NG_047393.1	aminoglycoside nucleotidyltransferase ANT(6)-Ia	STREPTOMYCIN
I	NODE_63_length_7203_cov_21.646269	3032	3574	+	sat4	1-543/543	100	100	ncbi	NG_048072.1	streptothricin N-acetyltransferase Sat4	STREPTOTHRICIN
K	NODE_41_length_3055_cov_6.701503	8	194	-	blaI_of_Z	1-187/381	49.08	95.72	ncbi	NG_047499.1	penicillinase repressor BlaI	BETA-LACTAM
K	NODE_41_length_3055_cov_6.701503	184	1941	-	blaR1	1-1758/1758	100	97.04	ncbi	NG_047539.1	beta-lactam sensor/signal transducer BlaR1	BETA-LACTAM
K	NODE_41_length_3055_cov_6.701503	2048	2893	+	blaZ	1-846/846	100	99.88	ncbi	NG_055999.1	penicillin-hydrolyzing class A beta-lactamase BlaZ	BETA-LACTAM
K	NODE_16_length_61663_cov_82.666277	35228	37147	+	tet(M)	1-1920/1920	100	100	ncbi	NG_048213.1	tetracycline resistance ribosomal protection protein Tet(M)	TETRACYCLINE
M	NODE_12_length_44293_cov_33.702554	28862	29242	+	blaI_of_Z	1-381/381	100	95.8	ncbi	NG_047499.1	penicillinase repressor BlaI	BETA-LACTAM
M	NODE_12_length_44293_cov_33.702554	26163	27008	-	blaPC1	1-846/846	100	99.29	ncbi	NG_052065.1	BlaZ family penicillin-hydrolyzing class A beta-lactamase PC1	BETA-LACTAM
M	NODE_12_length_44293_cov_33.702554	27115	28872	+	blaR1	1-1758/1758	100	98.46	ncbi	NG_047536.1	beta-lactam sensor/signal transducer BlaR1	BETA-LACTAM
M	NODE_5_length_173380_cov_33.727959	41352	43271	+	tet(M)	1-1920/1920	100	100	ncbi	NG_048213.1	tetracycline resistance ribosomal protection protein Tet(M)	TETRACYCLINE
N	NODE_76_length_8244_cov_11.231489	3784	4578	-	aph(3')-IIIa	1-795/795	100	100	ncbi	NG_047418.1	aminoglycoside O-phosphotransferase APH(3')-IIIa	AMIKACIN/KANAMYCIN
N	NODE_55_length_14557_cov_13.131947	13838	14218	-	blaI_of_Z	1-381/381	100	99.74	ncbi	NG_047499.1	penicillinase repressor BlaI	BETA-LACTAM
N	NODE_93_length_1902_cov_8.858028	603	1348	+	erm(B)	1-747/747	99.87	99.47	ncbi	NG_047801.1	23S rRNA (adenine(2058)-N(6))-methyltransferase Erm(B)	MACROLIDE
N	NODE_5_length_80395_cov_17.662406	76205	78211	-	mecA	1-2007/2007	100	99.95	ncbi	NG_047940.1	PBP2a family beta-lactam-resistant peptidoglycan transpeptidase MecA	METHICILLIN
N	NODE_5_length_80395_cov_17.662406	80068	80395	+	mecI_of_mecA	1-328/372	88.17	99.69	ncbi	NG_055650.1	mecA-type methicillin resistance repressor MecI	METHICILLIN
N	NODE_5_length_80395_cov_17.662406	78311	80068	+	mecR1	1-1758/1758	100	100	ncbi	NG_051163.1	beta-lactam sensor/signal transducer MecR1	METHICILLIN

N	NODE_76_length_8244_cov_11.231489	5210	6118	-	ant(6)-Ia	1-909/909	100	100	ncbi	NG_047393.1	aminoglycoside nucleotidyltransferase ANT(6)-Ia	STREPTOMYCIN
N	NODE_76_length_8244_cov_11.231489	4671	5213	-	sat4	1-543/543	100	100	ncbi	NG_048072.1	streptothricin N-acetyltransferase Sat4	STREPTOTHRICIN
N	NODE_62_length_12311_cov_13.623605	9630	10127	-	dfrG	1-498/498	100	100	ncbi	NG_047756.1	trimethoprim-resistant dihydrofolate reductase DfrG	TRIMETHOPRIM
O	NODE_49_length_7564_cov_27.224418	3170	3964	-	aph(3')-IIIa	1-795/795	100	100	ncbi	NG_047418.1	aminoglycoside O-phosphotransferase APH(3')-IIIa	AMIKACIN/KANAMYCIN
O	NODE_7_length_97650_cov_41.917701	96551	96931	-	blaI_of_Z	1-381/381	100	95.54	ncbi	NG_047499.1	penicillinase repressor BlaI	BETA-LACTAM
O	NODE_64_length_2772_cov_4.081664	1111	1956	-	blaZ	1-846/846	100	96.57	ncbi	NG_055999.1	penicillin-hydrolyzing class A beta-lactamase BlaZ	BETA-LACTAM
O	NODE_50_length_7549_cov_24.066289	2559	3206	-	catA7	1-648/648	100	100	ncbi	NG_047568.1	type A-7 chloramphenicol O-acetyltransferase	CHLORAMPHENICOL
O	NODE_50_length_7549_cov_24.066289	6614	7351	+	erm(B)	1-738/738	100	100	ncbi	NG_047794.1	23S rRNA (adenine(2058)-N(6))-methyltransferase Erm(B)	MACROLIDE
O	NODE_49_length_7564_cov_27.224418	4596	5504	-	ant(6)-Ia	1-909/909	100	100	ncbi	NG_047393.1	aminoglycoside nucleotidyltransferase ANT(6)-Ia	STREPTOMYCIN
O	NODE_49_length_7564_cov_27.224418	4057	4599	-	sat4	1-543/543	100	100	ncbi	NG_048072.1	streptothricin N-acetyltransferase Sat4	STREPTOTHRICIN
P	NODE_1_length_1093950_cov_19.619101	500450	501244	-	aph(3')-IIIa	1-795/795	100	100	ncbi	NG_047418.1	aminoglycoside O-phosphotransferase APH(3')-IIIa	AMIKACIN/KANAMYCIN
P	NODE_3_length_486508_cov_21.174392	367930	368310	-	blaI_of_Z	1-381/381	100	99.74	ncbi	NG_047499.1	penicillinase repressor BlaI	BETA-LACTAM
P	NODE_3_length_486508_cov_21.174392	370195	371040	+	blaPC1	1-846/846	100	99.41	ncbi	NG_052065.1	BlaZ family penicillin-hydrolyzing class A beta-lactamase PC1	BETA-LACTAM
P	NODE_3_length_486508_cov_21.174392	368300	370057	-	blaR1	1-1758/1758	100	99.94	ncbi	NG_047539.1	beta-lactam sensor/signal transducer BlaR1	BETA-LACTAM
P	NODE_1_length_1093950_cov_19.619101	506035	506780	-	erm(B)	1-747/747	99.87	99.47	ncbi	NG_047801.1	23S rRNA (adenine(2058)-N(6))-methyltransferase Erm(B)	MACROLIDE
P	NODE_3_length_486508_cov_21.174392	230016	232022	+	mecA	1-2007/2007	100	99.95	ncbi	NG_047940.1	PBP2a family beta-lactam-resistant peptidoglycan transpeptidase MecA	METHICILLIN
P	NODE_3_length_486508_cov_21.174392	227788	228159	-	mecI_of_mecA	1-372/372	100	99.73	ncbi	NG_055650.1	mecA-type methicillin resistance repressor MecI	METHICILLIN
P	NODE_3_length_486508_cov_21.174392	228159	229916	-	mecR1	1-1758/1758	100	100	ncbi	NG_051163.1	beta-lactam sensor/signal transducer MecR1	METHICILLIN
P	NODE_1_length_1093950_cov_19.619101	501876	502784	-	ant(6)-Ia	1-909/909	100	100	ncbi	NG_047393.1	aminoglycoside nucleotidyltransferase ANT(6)-Ia	STREPTOMYCIN
P	NODE_1_length_1093950_cov_19.619101	501337	501879	-	sat4	1-543/543	100	100	ncbi	NG_048072.1	streptothricin N-acetyltransferase Sat4	STREPTOTHRICIN
P	NODE_1_length_1093950_cov_19.619101	509548	510045	+	dfrG	1-498/498	100	100	ncbi	NG_047756.1	trimethoprim-resistant dihydrofolate reductase DfrG	TRIMETHOPRIM
Q	NODE_146_length_3396_cov_4.632915	508	1302	+	aph(3')-IIIa	1-795/795	100	100	ncbi	NG_047418.1	aminoglycoside O-phosphotransferase APH(3')-IIIa	AMIKACIN/KANAMYCIN
Q	NODE_7_length_50598_cov_13.812050	1850	2230	+	blaI_of_Z	1-381/381	100	99.74	ncbi	NG_047499.1	penicillinase repressor BlaI	BETA-LACTAM
Q	NODE_7_length_50598_cov_13.812050	487	1860	+	blaR1	385-1758/1758	78.16	100	ncbi	NG_047539.1	beta-lactam sensor/signal transducer BlaR1	BETA-LACTAM
Q	NODE_164_length_2183_cov_3.437743	888	1633	+	erm(B)	1-747/747	99.87	99.47	ncbi	NG_047801.1	23S rRNA (adenine(2058)-N(6))-methyltransferase Erm(B)	MACROLIDE
Q	NODE_185_length_1542_cov_9.159011	1	1542	-	mecA	314-1855/2007	76.83	100	ncbi	NG_047938.1	PBP2a family beta-lactam-resistant peptidoglycan transpeptidase MecA	METHICILLIN
Q	NODE_117_length_6383_cov_5.126758	1825	2196	+	mecI_of_mecA	1-372/372	100	99.73	ncbi	NG_055650.1	mecA-type methicillin resistance repressor MecI	METHICILLIN

Q	NODE_117_length_6383_cov_5.126758	68	1825	+	mecR1	1-1758/1758	100	100	ncbi	NG_051163.1	beta-lactam sensor/signal transducer MecR1	METHICILLIN
Q	NODE_146_length_3396_cov_4.632915	1	415	+	sat4	129-543/543	76.43	100	ncbi	NG_048070.1	streptothricin N-acetyltransferase Sat4	STREPTOTHRICIN
Q	NODE_202_length_1279_cov_7533.359375	364	1279	-	tet(K)	465-1380/1380	66.38	100	ncbi	NG_048200.1	tetracycline efflux MFS transporter Tet(K)	TETRACYCLINE
Q	NODE_158_length_2463_cov_5.922517	1995	2463	+	dfrG	1-469/498	94.18	100	ncbi	NG_047756.1	trimethoprim-resistant dihydrofolate reductase DfrG	TRIMETHOPRIM
T	NODE_102_length_3966_cov_7.122167	2743	3537	-	aph(3')-IIIa	1-795/795	100	100	ncbi	NG_047418.1	aminoglycoside O-phosphotransferase APH(3')-IIIa	AMIKACIN/KANAMYCIN
T	NODE_134_length_1200_cov_2.375582	784	1164	+	blaI_of_Z	1-381/381	100	99.74	ncbi	NG_047499.1	penicillinase repressor BlaI	BETA-LACTAM
T	NODE_134_length_1200_cov_2.375582	1	794	+	blaR1	965-1758/1758	45.16	99.87	ncbi	NG_047539.1	beta-lactam sensor/signal transducer BlaR1	BETA-LACTAM
T	NODE_29_length_37806_cov_11.161974	555	1300	-	erm(B)	1-747/747	99.87	99.47	ncbi	NG_047801.1	23S rRNA (adenine(2058)-N(6))-methyltransferase Erm(B)	MACROLIDE
T	NODE_12_length_51153_cov_14.041410	68	2074	+	mecA	1-2007/2007	100	99.95	ncbi	NG_047940.1	PBP2a family beta-lactam-resistant peptidoglycan transpeptidase MecA	METHICILLIN
T	NODE_90_length_6201_cov_6.270991	4340	4711	-	mecI_of_mecA	1-372/372	100	99.73	ncbi	NG_055650.1	mecA-type methicillin resistance repressor MecI	METHICILLIN
T	NODE_90_length_6201_cov_6.270991	4711	6201	-	mecR1	268-1758/1758	84.81	100	ncbi	NG_051163.1	beta-lactam sensor/signal transducer MecR1	METHICILLIN
T	NODE_107_length_3249_cov_4.715567	2127	3035	+	ant(6)-Ia	1-909/909	100	100	ncbi	NG_047393.1	aminoglycoside nucleotidyltransferase ANT(6)-Ia	STREPTOMYCIN
T	NODE_102_length_3966_cov_7.122167	3630	3884	-	sat4	289-543/543	46.96	100	ncbi	NG_048070.1	streptothricin N-acetyltransferase Sat4	STREPTOTHRICIN
T	NODE_29_length_37806_cov_11.161974	4068	4565	+	dfrG	1-498/498	100	100	ncbi	NG_047756.1	trimethoprim-resistant dihydrofolate reductase DfrG	TRIMETHOPRIM
X	NODE_24_length_19387_cov_29.540654	17970	18350	-	blaI_of_Z	1-381/381	100	100	ncbi	NG_047499.1	penicillinase repressor BlaI	BETA-LACTAM
X	NODE_40_length_1680_cov_5.960721	772	1617	+	blaPC1	1-846/846	100	99.41	ncbi	NG_052065.1	BlaZ family penicillin-hydrolyzing class A beta-lactamase PC1	BETA-LACTAM
X	NODE_24_length_19387_cov_29.540654	18340	19322	-	blaR1	776-1758/1758	55.92	99.9	ncbi	NG_047539.1	beta-lactam sensor/signal transducer BlaR1	BETA-LACTAM
X	NODE_3_length_190127_cov_48.878111	67268	68013	-	erm(B)	1-747/747	99.87	99.47	ncbi	NG_047801.1	23S rRNA (adenine(2058)-N(6))-methyltransferase Erm(B)	MACROLIDE
X	NODE_3_length_190127_cov_48.878111	63486	64352	-	aadE	1-867/867	100	100	ncbi	NG_047378.1	aminoglycoside 6-adenylyltransferase AadE	STREPTOMYCIN
X	NODE_11_length_85039_cov_47.280184	29619	31538	+	tet(M)	1-1920/1920	100	100	ncbi	NG_048252.1	tetracycline resistance ribosomal protection protein Tet(M)	TETRACYCLINE
X	NODE_3_length_190127_cov_48.878111	72004	73923	-	tet(O)	1-1920/1920	100	100	ncbi	NG_048255.1	tetracycline resistance ribosomal protection protein Tet(O)	TETRACYCLINE

Supplementary Table 1: Antibiotic resistance genes and products present in *S. pseudintermedius* isolates identified using ABRICATE. A threshold of >90% sequence identity was used to determine a good match between the database genes and hits within the genome sequences.

Supplementary figure 1

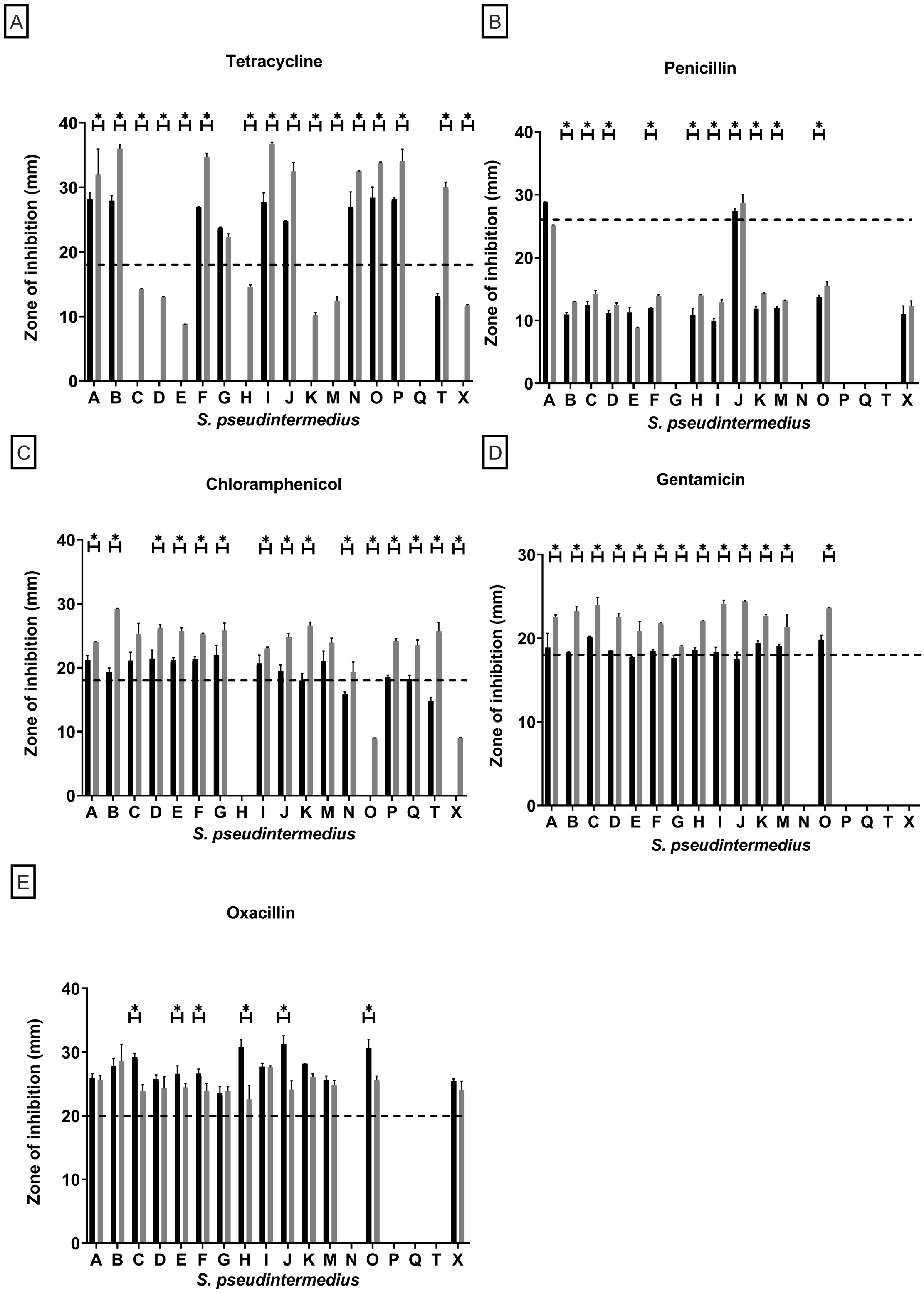

Supplementary figure 1: Graph A-E shows the zone diameter in mm of tetracycline, penicillin, chloramphenicol gentamicin and oxacillin respectively against 18 isolates of *S. pseudintermedius* in MHB (black bars) and with a sub lethal concentration of manuka honey, 5% (w/v) (grey bars). The horizontal dashed line represents the clinical breakpoint according to the EUCAST guidelines, everything below the line is deemed to be resistant. Those isolates showing a significant increase in sensitivity are marked with an asterisk (p= 0.05). Bars show median values and error bars represent 95% confidence limit.

Supplementary figure 2

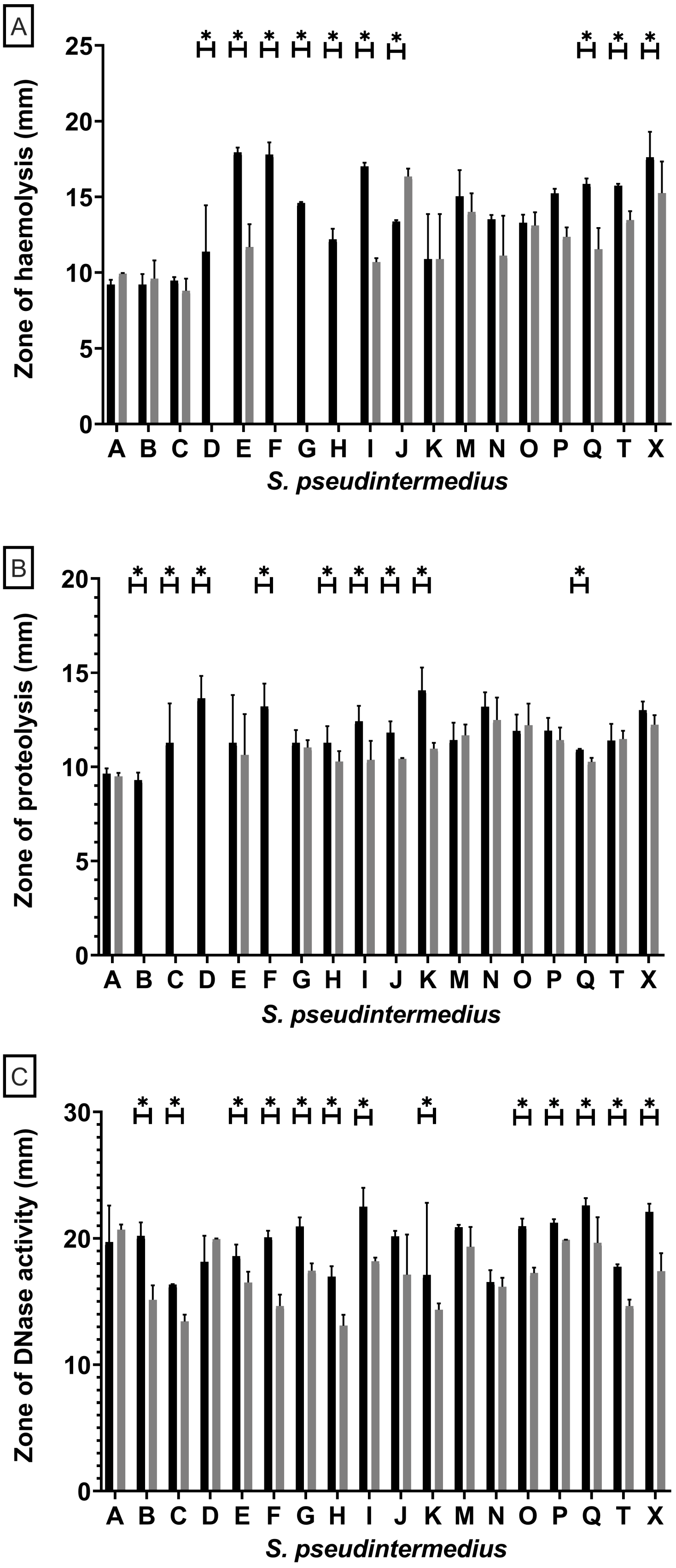
A
Zone of haemolysis (mm)
0
5
10
15
20
25
A B C D E F G H I J K M N O P Q T X
S. pseudintermedius
B
Zone of proteolysis (mm)
0
5
10
15
20
A B C D E F G H I J K M N O P Q T X
S. pseudintermedius
C
Zone of DNase activity (mm)
0
10
20
30
A B C D E F G H I J K M N O P Q T X
S. pseudintermedius

Supplementary figure 2: Manuka honey is able to inhibit virulence factor production by *S. pseudintermedius*. Haemolytic (A) proteolytic (B) and DNase (C) activity of the 18 *S. pseudintermedius* isolates in the absence (black bars) or presence (grey bars) of a sub lethal (5% w/v) concentration of manuka honey. Those isolates showing a significant ($p \leq 0.05$) change in activity are marked with an asterisk.

Bars show median values and error bars represent 95% confidence limit.
